# Synthesis of DOPO-Based Phosphorus-Nitrogen Containing Hyperbranched Flame Retardant and Its Effective Application for Poly(ethylene terephthalate) via Synergistic Effect

**DOI:** 10.3390/polym15030662

**Published:** 2023-01-28

**Authors:** Hossamaldin Ahmed Omer Abdalrhem, Yueyue Pan, Hongda Gu, Xiang Ao, Xiaohuan Ji, Xiaoze Jiang, Bin Sun

**Affiliations:** 1 State Key Laboratory for Modification of Chemical Fibers and Polymer Materials, College of Materials Science and Engineering, Donghua University, Shanghai 201620, China; 2 Changshu Polyester Co., Ltd., Changshu 215535, China

**Keywords:** P-N synergistic flame retardant, flame retardant mechanism, blending, PET, hyperbranched polyester

## Abstract

To obtain industrialized poly(ethylene terephthalate) (PET) composites with highly efficient flame retardancy, a phosphorus-nitrogen (P-N) containing hyperbranched flame retardant additive was synthesized by 9,10-dihydro-9-oxa-10-phospho-phenanthrene-butyric acid (DDP) and tris(2-hydroxyethyl) isocyanurate (THEIC) through high temperature esterification known as hyperbranched DDP-THEIC (hbDT). The chemical structure of the synthesized hbDT was determined by FTIR, ^1^H NMR, ^13^C NMR, and GPC, etc. Subsequently, hbDT/PET composites were prepared by co-blending, and the effects of hbDT on the thermal stability, flame retardancy, combustion performance, and thermal degradation behavior of PET were explored to deeply analyze its flame retardant mechanism. The test results showed that hbDT was successfully synthesized, and that hbDT maintained thermal stability well with the required processing conditions of PET as retardant additives. The flame retardant efficiency of PET was clearly improved by the addition of hbDT via the synergistic flame-retardant effect of P and N elements. When the mass fraction of flame retardant was 5%, the LOI of the hbDT/PET composite increased to 30.2%, and the vertical combustion grade reached UL-94 V-0. Compared with pure PET, great decreased total heat release (decreased by 16.3%) and peak heat release rate (decreased by 54.9%) were exhibited. Finally, the flame retardant mechanism of hbDT/PET was supposed, and it was confirmed that retardant effect happened in both the gas phase and condensed phase. This study is expected to provide a new idea for the development of low toxic, environment-friendly and highly efficient flame retardant additive for polyesters in an industry scale.

## 1. Introduction

Poly(ethylene terephthalate) (PET) is one of the most widely used and highly yielding thermoplastic polymers in the world, and is significantly applied in the fields of construction, automobiles, textiles, etc. [1,2]. However, the extremely flammable characteristic of PET poses a safety hazard and limits their usage in some exclusive fire protection applications [3]. Therefore, it is of great significance to develop non-toxic, highly efficient flame retardant PET products.

The flame retardant modification of PET mainly includes copolymerization, surface treatment and the bulk-additive method [3,4] The bulk-additive method is the physical mixing of flame retardants with polymer matrix, and is particularly suitable for scaled up industrial production. So far, the most common flame retardant additives used in PET can be classified into inorganic [5,6], halogen-containing [7,8]**,** and phosphorus-containing flame retardants [9,10]. However, inorganic flame retardants have poor compatibility with the polyesters and halogen flame retardants will release harmful and toxic substances by pyrolysis. Phosphorus-containing flame retardants have become a superior choice with their excellent flame retardancy and relatively environment friendliness. Moreover, “binary flame retardant”, usually phosphorus with another element can improve their flame retardancy due to the synergistic effect [11,12]. As a traditional flame retardant, nitrogen-containing flame retardant is non-toxic and plays a positive role in suppressing the smoke generated by combustion. Therefore, phosphorus-nitrogen (P-N) synergy can further improve the flame-retardant efficiency, where P-containing segments promote charring as the dehydrating agent in the condensed phase, while N-containing segments make main contribution in gas phase [13].

The P-N synergetic flame retardants used in polyesters are mainly classified into three categories based on the molecular weight and structure: small molecules [14], polymeric linear molecules [15]**,** and hyperbranched polymers [16]. In recent years, hyperbranched flame retardants have gained a lot of attention due to their low crystallinity, relative low viscosity with non-entangled structures (compared to linear polymers), high solubility, and high activity with multi-functional end groups (in comparison to small molecules). In previous research, hyperbranched polymers have been applied as rheology and crystalline modifiers in PET materials to promote the processability [17,18], because when the relative molecular weight of PET is too high, PET exhibits high characteristic viscosity**,** high crystallinity and poor melt-flowability, leading to difficulties in molding and processing. However, the addition of hyperbranched polymers to achieve excellent flame retardancy always results in excessive reduction of the viscosity and deterioration of glass transition temperature (Tg) of PET [19]. Therefore, how to balance the necessity to improve the processing properties of PET melt with the demand to maintain good flame retardancy is an urgent issue to be solved.

9,10-dihydro-9-oxa-10-phosphorus-10-oxide (DOPO) and its derives are one of the most outstanding commercial phosphorus-containing flame retardants used in polymers, due to their high thermal stability, strong chemical durability, and minimal side effects on the mechanical properties of polymer matrix [20]. Moreover, DOPO has highly reactive P-H bond and DOPO derives have other reactive terminal functional groups (such as hydroxyl, carboxyl, amino and anhydride groups, etc.). With the help of bond-to-bond chemical reactions, nitrogen-containing flame retardants can be grafted to DOPO structures to produce new high-efficiency P-N synergistic flame retardants. For the influence of Tg of polymers, Tg appears to increase with both chain stiffness and bulkier side groups [21,22]. In recent decades, some DOPO derivatives modified polyesters show maintained or increased Tg [9,23,24], because the bulky rigid DOPO groups impede the mobility of chain segments.

In this work, a new DOPO derive-based P-N co-efficient flame retardant hyperbranched DDP-THEIC (hbDT) was synthesized by 9,10-Dihydro-10-(2,3-dicarboxypropyl)-9-oxa-10-phosphaphenanthrene 10-oxide (DDP) and tris (2-hydroxyethyl) isocyanurate (THEIC). The detailed chemical structures have been depicted by FTIR, NMR and GPC. The hbDT/PET composites with different hbDT loading amounts were obtained by melt blending method, and the effects of hbDT on the thermal stability, flame retardancy, burning behavior and thermal degradation behavior of PET were analyzed. Finally, the synergistic flame retardant mechanism of the DDP and THEIC with the hyperbranched structure was proposed.

## 2. Materials and Methods

### 2.1. Materials

9,10-Dihydro-10-(2,3-dicarboxypropyl)-9-oxa-10-phosphaphenanthrene 10-oxide (DDP) and tris (2-hydroxyethyl) isocyanurate (THEIC) were supplied from Weng Jiang Reagent (industrial grade, Guangzhou, China). P-toluenesulfonic acid (p-TSA) and N,N-dimethylformamide (DMF) were acquired from Sinopharm Chemical Reagent Co., Ltd. (analytical grade, Shanghai, China). Bright polyethylene terephthalate (PET) was purchased from Sinopec Yizheng Chemical Fiber Co., Ltd. (intrinsic viscosity = 0.64, industrial grade, Yangzhou, China). All of the chemicals were used as received without additional purification.

### 2.2. Synthesis of hbDT

**Scheme 1:**
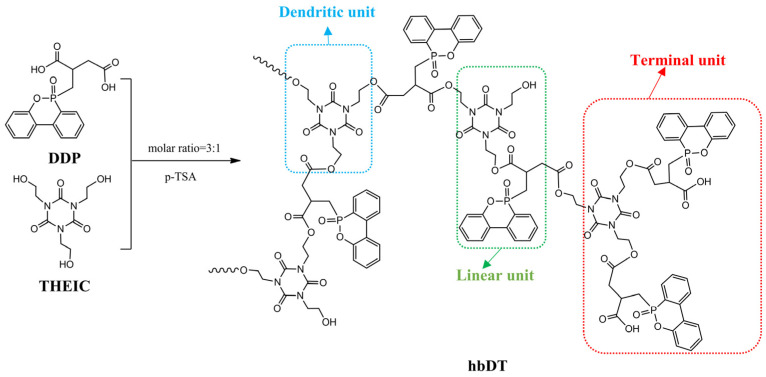
Schematic preparation and structural formula of hbDT.

Synthesis of hbDT was carried out in a 500 mL three-necked flask equipped with a reflux condenser, a mechanical stirrer and a N_2_ inlet valve. DDP (104.4 g, 0.3 mol), THEIC (26.1 g, 0.1 mol), and p-TSA (0.172 g, 0.001 mol, as catalyst) were introduced with 250 mL DMF as solvent. The mixture in the reactor was immersed in an oil bath at the temperature of 120 °C with vigorous stirring speed of 300 rpm for 3 h. Before and during the synthesis process, nitrogen was ventilated constantly into the reactor to remove the residual air and moisture. After the reaction, 130 mL deionized water was added slowly in drops to the reaction solvent and white precipitates were obtained. The precipitates were filtered, thoroughly cleaned with plenty of water, and then treated with ethanol before being vacuum-dried at 50 °C for 12 h. The obtained white powders were labeled as hbDT. The schematic preparation and structural formula of hbDT are illustrated in Scheme 1.

### 2.3. Preparation of hbDT/PET Composites

The PET slices and hbDT powders should be pretreated in the vacuum oven to remove the moisture. The PET was first dried at 80 °C for 18 h, and then heated up to 130 °C for 12 h with vacuum pressure of 0.1 Mpa, while the hbDT was dried at 50 °C for 12 h. The pretreated PET and hbDT was mechanically mixed with different complex contents (0 wt%, 2 wt%, 5 wt% and 20 wt% of hbDT), thereafter, the mixtures were introduced to a two-screw extruder (model SHJ20, Nanjing Jieya Extrusion Equipment Co., Ltd., China, D = 21.7 mm, L/D = 30) at 260 °C and at the screw speed of 400 rpm. Finally, the extruded products were injected using an injection machine. The sample chips were labeled as PET, 2%hbDT/PET, 5%hbDT/PET, and 20%hbDT/PET, in which 2%, 5%, and 20% represented for mass fraction of hbDT. The morphology and the element information were illustrated in Figure S2 in the supporting information.

### 2.4. Characterization

Structural Characterization. A Nicolet iS20 infrared spectrometer (ThermoFisher, Waltham, MA, USA) was used to record Fourier transform infrared (FTIR) spectroscopy from 400 to 4000 cm^−1^ with a spectral resolution of 4 cm^−1^ and 32 scans. The samples were ground and mixed with KBr powder to make test tablets. The NMR measurements were carried out on a 400 AVANCE spectrometer (Bruker, Karlsruhe, Germany) operating at 400 MHz for ^1^H and at 151 MHz for ^13^C NMR spectra, with deuterated dimethyl sulfoxide (DMSO-d_6_) as solvent and tetramethylsilane (TMS) as internal standard. Gel permeation chromatography (GPC) measurement was carried out using a Viscotek GPC/SEC apparatus (Malvern, UK) with DMF as the mobile phase at the flow rate of 1 mL/min at 40 °C, and polystyrene was used as the standard specimen.

Degree of branching (DB). In general, hyperbranched polymers are composed of linear (L), dendritic (D) and terminal (T) units. The DB is a measurement on the content of branches in the molecular structure and calculated by the quantitative ^13^C NMR spectra (with detailed information of Figure S1 in supporting information). Quantitative ^13^C NMR spectra were obtained using inverse gated decoupling mode with a pulse delay of 3 s. DB of the hyperbranched polymers has been defined as follows [25]:

DB = (D + T)/(D + T + L) (1)

where D, T, and L refer to the numbers of dendritic, terminal, and linear units, respectively.

Thermogravimetric analysis (TGA) was conducted by a TG 209 F1 thermo analyzer (Netzsch, Selb, Germany). A sample weighing between 5 and 10 mg was placed in a ceramic crucible and heated from room temperature to 700 °C at a heating rate of 20 °C·min^−1^ in an alternate atmosphere of nitrogen and air. Differential scanning calorimetry (DSC) was conducted on a DSC Q2000 (TA Instruments Inc., New Castle, DE, USA) under nitrogen. Tg was measured at the mid-point of the inflection curve from the second heating cycle at a heating rate of 10 °C·min^−1^. The thermodegradation process of the composite was observed using in situ FTIR spectra with a spectral resolution of 4 cm^−1^ at temperatures between 400 and 700 °C with a heating rate of 10 °C/min in N_2_ atmosphere. The surface property of char layer after combustion was conducted by a HITACHI S-4800 scanning electron microscope (Hitachi, Japan). Before observation, all sample surfaces were coated with gold.

The limiting oxygen index (LOI) were recorded using a ZR-1 oxygen index meter (Qingdao Zhongbang, China) in the manner of GB/T 2406.2-2009 standard, and the samples were prepared with dimension size of 100 mm × 10 mm × 4 mm. The UL-94 vertical burning test was performed on a LX-8820D-type vertical burning test instrument depending on the GB/T 2408-2008 testing procedure. The dimension of the sample was 125 mm × 13 mm × 3.2 mm. During LOI and vertical burning tests, each sample was measured 5 times and the average results were taken. The cone calorimeter method test was carried out using a cone calorimeter (Standard, Fire Test Technology Co., Ltd., West Sussex, UK) according to GB/T16172-2007/ISO5660-1:2002 standard. The samples used in the tests were at the size of 100 mm × 100 mm × 3 mm, and the measurement was performed at a heat radiation power of 50 kW·m^−2^. The HP5890SA Py-GC-MS spectrometer was used to measure the pyrolysis-gas chromatogram-mass (Py-GC-MS) analysis of pure PET and hbDT/PET composites at a pyrolysis temperature of 500 °C, holding time 20 s and heating rate of 200 °C·ms^−1^.

## 3. Results and Discussion

Herein, A_2_ + B_3_ condensation method [26] was used to synthesize hyperbranched polymers containing phosphorus-nitrogen molecules. The obtained hyperbranched polymers were used as additives into PET matrix to study the effect of chemical composition and addition amounts on the flame retardancy of hbDT/PET composites.

### 3.1. Structure Characterization of hbDT

**Figure 1:**
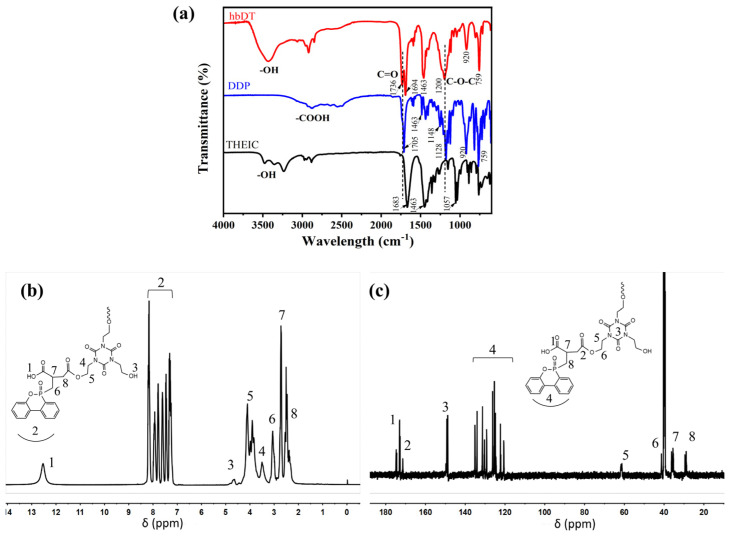
(**a**) FTIR spectra of DDP, THEIC and hbDT, (**b**) ^1^H NMR and (**c**) ^13^C NMR spectra of hbDT.

The structure of hbDT was characterized by FTIR, ^1^H-NMR and ^13^C-NMR. In Figure 1a, the FTIR spectra of DDP, THEIC monomer and final product hbDT are presented. From the FTIR spectrum of hbDT, it was observed that the stretching vibration absorption peak of –OH derived from THEIC at 3400~3500 cm^−1^ and –COOH derived from DDP at 2900~3100 cm^−1^ were weakened due to esterification between DDP and THEIC. Furthermore, the typical absorption peak of C-O single bonding from THEIC at 1057 cm^−1^ disappeared, and a new peak at 1200 cm^−1^ which can be attributed to the symmetrical stretching vibration peak of C-O-C in the ester bond appeared [27]. In addition, compared with the C=O stretching vibration absorption peak at 1705 cm^−1^ of DDP and 1683 cm^−1^ of THEIC, the red-shift of C=O stretching vibration at 1736 cm^−1^ in hbDT spectrum can be attributed to the new C=O double bond in ester bond due to the esterification reaction [28]. Therefore, the FTIR results mentioned above showed the high intensity absorption peaks of C-O-C and C=O in the hbDT spectrum, which indicated the successful esterification between DDP and THEIC to produce hbDT molecule. The molecular weight and molecular weight distribution of hbDT were determined by GPC. The number-average molecular weight (M_n_), weight-average molecular weight (M_w_), and polydispersity index (PDI) of hbDT were 6700 g·mol^−1^, 11,100 g·mol^−1^ and 1.66, respectively.

The ^1^H-NMR and ^13^C-NMR spectra shown in Figure 1b,c were provided with more information to confirm the chemical structure of hbDT. In Figure 1b, ^1^H-NMR spectrum are d_(H1)_ = 12.5 ppm, d_(H2)_ = 7.2–8.3 ppm, d_(H3)_ = 4.7 ppm, d_(H4,5)_ = 3.5–4.1 ppm, d_(H6)_ = 2.9–3.1 ppm, d_(H7)_ = 2.7 ppm, d_(H8)_ = 2.3–2.5 ppm. In Figure 1c, ^13^C-NMR spectrum are d_(C1,2)_= 171.2–174.8 ppm, d_(C3)_ = 148.7–149.7 ppm, d_(C4)_ = 120.5–135.1 ppm, d_(C5)_ = 61.2–61.7 ppm, d_(C6)_ = 38.5–41.4 ppm, d_(C7)_ = 35.3–36.2 ppm, d_(C8)_ = 28.9–29.5 ppm. From the FTIR results and the NMR analysis, it is clearly seen that carboxylate groups generated by the reaction between DDP and THEIC were presented in the product. The degree of branching (DB) of obtained hbDT is 0.65. The results suggested the successful synthesis of hbDT. In addition, the phosphorus and nitrogen contents of hbDT were 7.38 wt% and 3.14 wt% tested by Inductively Coupled Plasma (ICP).

### 3.2. Thermal Performance of hbDT

**Figure 2:**
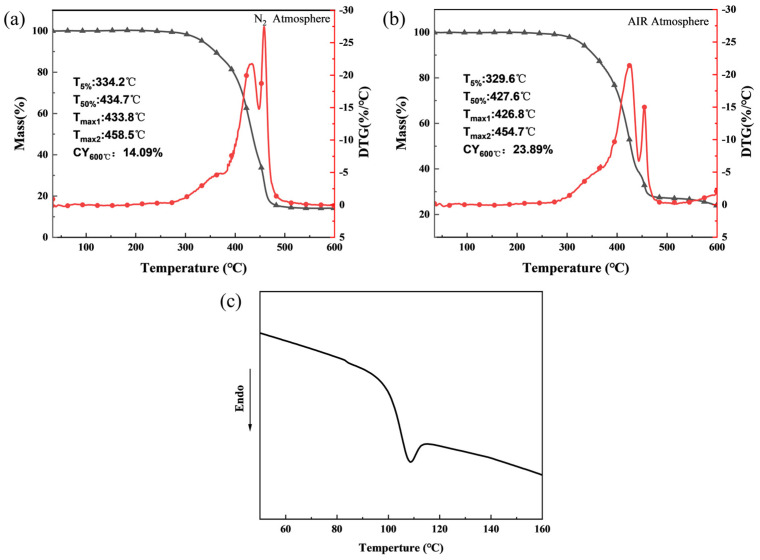
TG and DTG curves of hbDT (**a**) under N_2_ atmosphere and (**b**) under air atmosphere, (**c**) DSC curve of hbDT in N_2_.

The thermal stability of hbDT was investigated by TGA, the corresponding TGA and DTG curves in nitrogen and air are exhibited in Figure 2a,b, respectively. The results showed that although under different atmosphere, hbDT had similar Mass-Temperature curves that both presented two primary decomposition processes at 350~500 °C. In comparison to the processing temperature of PET (240–290 °C), the initial decomposition temperatures (T5%) of hbDT in N_2_ and in air were 334.2 °C and 329.6 °C, respectively. Therefore, hbDT can satisfy the processing criteria of PET. However, compared with some details in temperature, the initial decomposition temperature (T_5%_), the half weight-loss temperature (T_50%_), and the maximal weight-loss temperature (T_max_) of hbDT in air were slightly lower than that in N_2_, which is easily understood that the oxygen in air facilitating the decomposition of flame retardants. Furthermore, the residue in air atmosphere (23.89%) at 600 °C was much higher, and almost two times than that in N_2_ atmosphere (14.09%). This might be attributed to the degradation of phosphorus-containing segments in hbDT produced phosphoric acid, polyphosphoric acid and its derivatives under oxygen. The resultants phosphoric products promoted the dehydration and carbonization of the hbDT [29]. More char residues were obtained through this process, and the stable char prohibited further decomposition of hbDT to make more residues left. Figure 2c is the DSC curve of hbDT, it could be found that the T_g_ value of hbDT was 105 °C, which was significantly higher than the temperature of pure PET.

### 3.3. Thermal Degradation Behavior of hbDT/PET Composites

**Figure 3:**
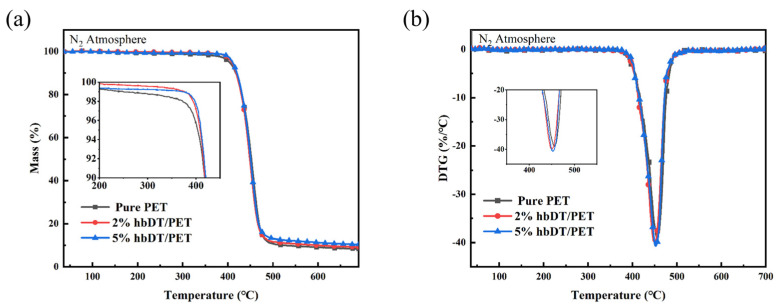
(**a**) TGA and (**b**) DTG curves of PET and different hbDT/PET composites in nitrogen atmosphere.

Previous research has shown that the combustion behavior of polymers is, to a certain extent, correlated with the thermal degradation process [30]. Herein, TGA was performed to study the thermal stability of hbDT/PET composites. Figure 3 depicts the temperature dependent weight loss as well as the derivative of the weight loss curves of PET and hbDT/PET composites in nitrogen. The characteristic temperatures of thermal degradation including the temperature at mass loss of 5% (T_5%_), the temperature at maximum weight-loss rate (T_max_) and the char yield (CY) at 700 °C are summarized in Table 1.

Compared with pure PET, the T_5%_ values of different hbDT/PET composites had a slight increase with the increase of hbDT in nitrogen atmosphere in Figure 3a. These results indicated that the addition of hbDT maintained the onset temperature for decomposition of PET, which can be attributed to the stable aromatic ring structure in hbDT and the ensuing strong affinity with PET substrates. At the same time, the T_max_ values of hbDT/PET did not change much with the increasing hbDT content of the composites, indicating that the addition of hbDT had no significant effect on the thermal decomposition process of PET. Based on the T_5%_ and T_max_ values, the hbDT is an excellent additive candidate to produce PET composites with good flame retardancy without influencing the characteristic thermal properties of PET. Additionally, as the char yield after the thermal decomposition is related to the LOI value of the composites, the char residue of the composite at high temperature directly affects the flame retardancy. At 700 °C, the char yield of pure PET was 8.33%, whereas the char yield of the composites after melt blending with hbDT rose from 9.23% to 10.97%, suggesting greater flame retardancy improvement.

Moreover, the increased char yield indicated the reaction between hbDT and PET matrix to promote the generation of char residues. In the initial stage of decomposition, the phosphorus groups in hbDT tended to produce phosphorus containing radicals and carbonized with PET backbone. Afterward, the phosphorus-rich char residue formed on the surface of PET matrix served as an effective layer to insulate heat and oxygen transfer, thus limiting further degradation of PET by increasing the combustion temperature.

**Figure 4:**
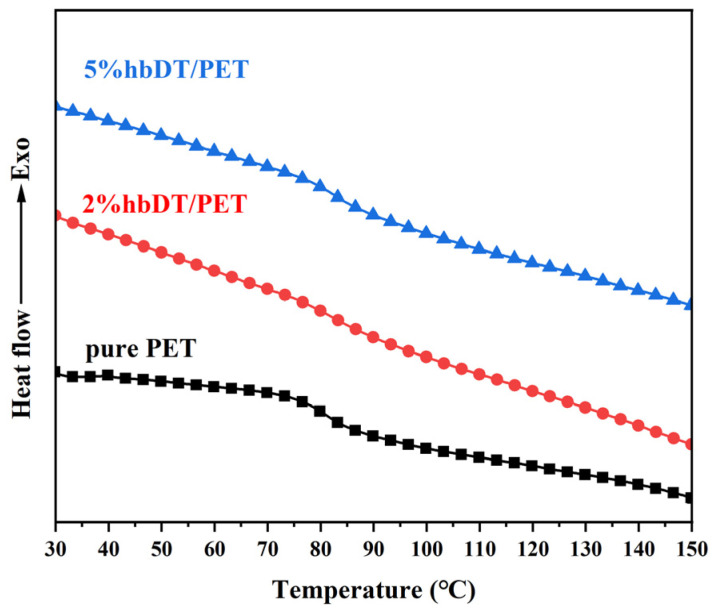
DSC curves of pure PET and different hbDT/PET composites.

The glass transition temperatures of PET and hbDT/PET composites were obtained from DSC. Figure 4 presents their DSC thermograms and the T_g_ values are listed in Table 1. From the DSC profile of PET, a T_g_ of 80.6 °C can be determined, and the Tg values of hbDT/PET composites with 2 wt% and 5 wt% hbDT were 81.8 °C and 83.3 °C, respectively. The increased T_g_ were attributed to the rigid structures of both THEIC and DDP, which is in consistent with the reported research [9,23,24].

### 3.4. Flame Retardance and Combustion Behavior of hbDT/PET Composites

**Figure 5:**
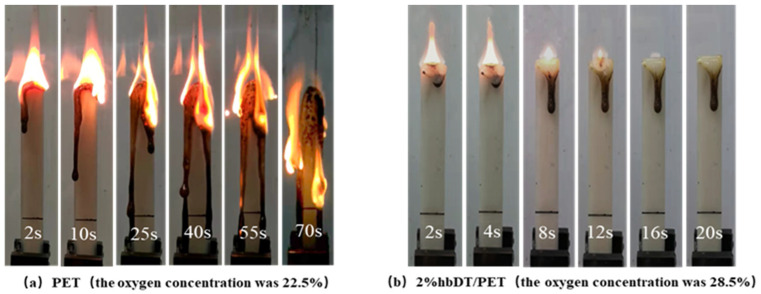
Digital photographs of (**a**) pure PET and (**b**) 2%hbDT/PET composite during the LOI test.

Melt dripping is a common phenomenon when thermoplastic polymers are burned, and it significantly accelerates fire propagation. Moreover, the LOI is the lowest oxygen content (vol. %) required to keep the sample burning, which is useful for investigating the fire resistance of materials. In general, flame retardants can promote the anti-dripping and fire resistance abilities. Therefore, to study the anti-dripping effect and fire resistant behaviors of hbDT in PET composites, the UL-94 vertical burning test and LOI test were performed. The corresponding results are presented in Table 2, and detailed processes of the LOI test are shown in Figure 5. In Table 2, compared with the no rating (NR, UL-94) of pure PET, the UL-94 rating of the hbDT/PET composite passed V-2 when the hbDT content reached to 2 wt%. In addition, with the increasing addition of hbDT additives to 5 wt%, the UL-94 rating had been further improved to V-0 rating. In the UL-94 vertical burning test process, although there were few melt droplets of 5%hbDT/PET chips after ignition, the droplets did not ignite the skimmed cotton again and achieved V-0 rating easily. The results clearly indicated that hbDT could rapidly form a char layer, showing excellent anti-dripping ability. It is clear from the LOI results that pure PET had a LOI value of 22.1%, indicating that it is particularly easy to combust. Meanwhile, when introducing the hbDT flame retardant, higher LOI values were obtained. As the hbDT additives increased from 2 wt% to 5 wt%, the LOI values increased from 28.9% to 30.2%, which was much higher than that of PET. Figure 5 showed the digital photographs of LOI test for pure PET and 2%hbDT/PET in detail. In Figure 5a, at an oxygen concentration of 22.5%, the PET burned fiercely after ignition and generated severe melt dripping. The heat from the top of the chips were carried away by the melt droplets, which continue to burn as they drop down. In contrast, the 2%hbDT/PET could be self-extinguished after burning 20 s as shown in Figure 5b, and the oxygen concentration increased to 28.5%. Though few molten droplets were still observed during combustion, the melt dripping phenomenon and LOI value had been improved obviously. These results indicated the excellent anti-dripping and fire resistance abilities of the new synthesized hbDT, and the performance can be further improved by increasing the contents of hbDT in hbDT/PET composites.

### 3.5. Combustion Properties of hbDT/PET

**Figure 6:**
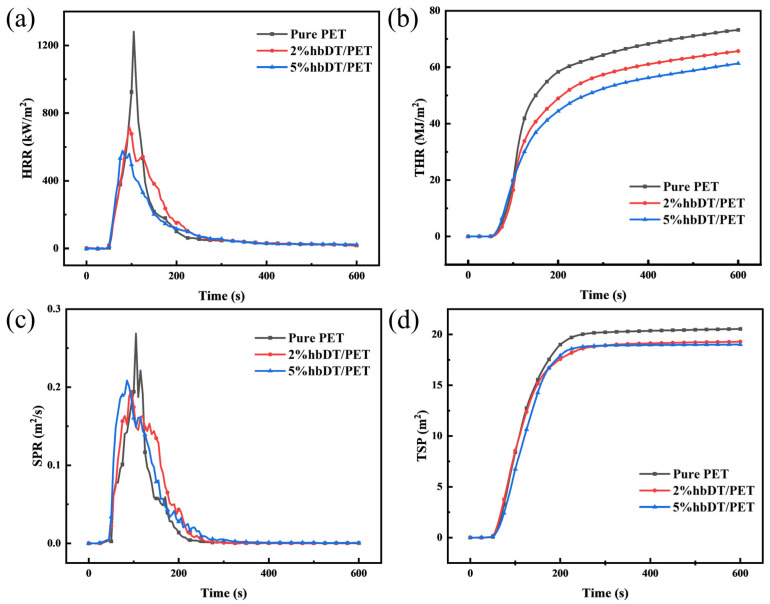
(**a**) Heat release rate (HRR), (**b**) total heat release (THR), (**c**) smoke production rate (SPR) and (**d**) total smoke production (TSP) curves of cone calorimeter test of pure PET and different hbDT/PET composites.

Cone calorimeter is the most effective lab-scale method to simulate real fire condition [31]. Hence, the cone calorimeter method was used in this work to investigate the comprehensive burning behaviors of hbDT/PET composites under real fire condition, and the results are illustrated in Figure 6 and Table 3. Heat release rate (HRR), especially peak heat release rate (pHRR), is regarded as the most important parameter for assessing the fire safety of polymer materials. The higher the HRR value, the faster the fire spreads during combustion [32]. As can be seen in Figure 6a and Table 3, the pHRR value of pure PET was 1281.6 kW·m^−2^. However, the pHRR of 2%hbDT/PET and 5%hbDT/PET decreased to 719.1 and 578.1 kW·m^−2^, which are 43.9% and 54.9% lower, respectively, compared to those of pure PET. In Figure 6b, the total heat release (THR) value of pure PET was 73.2 MJ·m^−2^, and the THR of 2%hbDT/PET and 5%hbDT/PET were 65.7 and 61.3 MJ·m^−2^, decreasing by 10.2% and 16.3%, respectively. These results indicated that the pure PET was a flammable polyester material, and the hbDT flame retardant improved the fire resistance of the hbDT/PET composites. Time to ignition (TTI) is the beginning time to ignite the materials, the shorter the time, the easier the ignition. As can be seen in Table 3, the introduction of hbDT first reduced the TTI of the hbDT/PET composites from 49 s to 42 s, and then increased to 45 s. This is probably because the initial thermal degradation temperature of the hbDT is lower than that of pure PET, resulting in an earlier start of degradation and ignition of the composites [33]. Meanwhile, with the increasing addition of flame retardants, the fire resistant effects of hbDT postponed the TTI. The dense smoke is one of the key factors that lead to death in a fire, thus the influence of hbDT on the smoke generation Smoke production rate (SPR) and total smoke production (TSP) of the PET composites were investigated in detail, and the results are presented in Figure 6c,d. Compared with pure PET, the SPR of hbDT/PET were significantly reduced. In Figure 6d and Table 3, the TSP of pure PET was 20.5 MJ·m^−2^, the TSP of 2%hbDT/PET and 5%hbDT/PET were 19.3 and 19.0 MJ·m^−2^, respectively, which were 5.9% and 7.3% less than that of PET. Therefore, hbDT might has certain smoke suppression advantages during combustion, which makes sense for safety in real applications. The effective heat of combustion (EHC) is the ratio of the real-time heat release to the real-time mass rate [34]. The EHC is the ratio of real-time heat release to real-time mass rate. It can exclude errors in THR values between different samples due to mass difference and other influencing factors, and also used to evaluate the gas phase dilution and condensed-phase barrier properties of flame retardants during combustion. Therefore, the reduction in the EHC is an important indicator of the gas phase flame retardant activity of a flame retardant. It is clearly observed in Table 3 that the average effective heat of combustion (AEHC) values of hbDT/PET composites were significantly lower than that of pure PET, which decreased from 17.4 MJ·kg^−1^ (pure PET) to 16.9 MJ·kg^−1^ (2%hbDT/PET) and 15.8 MJ·kg^−1^ (5%hbDT/PET), respectively. These results indicated that hbDT played an important role in the gas phase flame retardancy.

In Table 3, the char residue of pure PET was 2.6%. However, the char residues of 2%hbDT/PET and 5%hbDT/PET increased to 3.5 and 8.0 wt%, respectively. These results showed that hbDT could promote the formation of intumescent char layer on the surface of composite after ignition. The char covered the burning zone and acted as a physical barrier to isolate the transmission of flammable volatiles, oxygen, and external heat, which exhibited an excellent condensed-phase flame-retardant effect. More direct evidence was exhibited by SEM photo images in the following discussion.

### 3.6. Comparison of Flame Retardancy with Other PET Composites

To demonstrate the significance of this work, we compared it to other preciously reported flame retardant PET composites. The specific values of LOI values, and the decrease percentage of pHRR (ΔpHRR) and THR (ΔTHR) are listed in Table S1. In general, as these three values increase, the histogram increases, and the flame retardancy and fire safety of the PET composites are improved. It is clear observed from the comparison results that hbDT/PET have relatively high flame retardant efficiency. This kind of hbDT additive exhibited potential application prospects in the field of industrialized flame retardant PET composites.

### 3.7. Flame Retardant Mechanism of hbDT/PET

**Figure 7:**
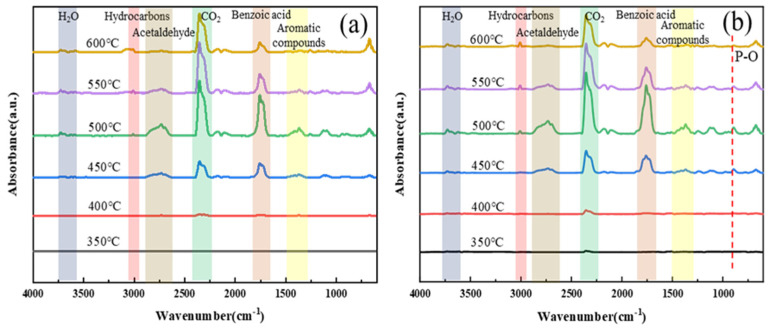
FTIR spectra of pyrolysis gases at different temperature for (**a**) PET and (**b**) 20 wt%hbDT/PET in TG-IR tests.

To explore the mechanism of hbDT in PET, the thermogravimetric analysis with infrared spectroscopy (TG-IR) was used to analyze gaseous products during the thermal decomposition process of pure PET and 20%hbDT/PET composite, and the spectra are presented in Figure 7a,b, respectively. The results showed that the main gaseous products of hbDT/PET were similar to those of PET, exhibiting characteristic bands of H_2_O at 3400–3900 cm^−1^, hydrocarbons around 3000 cm^−1^, acetaldehyde at 2600–2900 cm^−1^, CO_2_ at 2200–2400 cm^−1^, benzoic acid at 1640–1880 cm^−1^, and aromatic compounds at 1270–1480 cm^−1^ [35]. Meanwhile, for hbDT/PET in Figure 7b, in addition to the absorption peaks generated by the decomposition of PET, a new absorption peak emerged at 897 cm^−1^, which was ascribed to P-O bond stretching vibration peak by the thermal degradation of hbDT. This result indicated that the DOPO structure of hbDT was broken down to P-containing radicals and volatilized into the gas phase [36], which can trap combustible radicals in gas phase to resist the fire spread.

**Figure 8:**
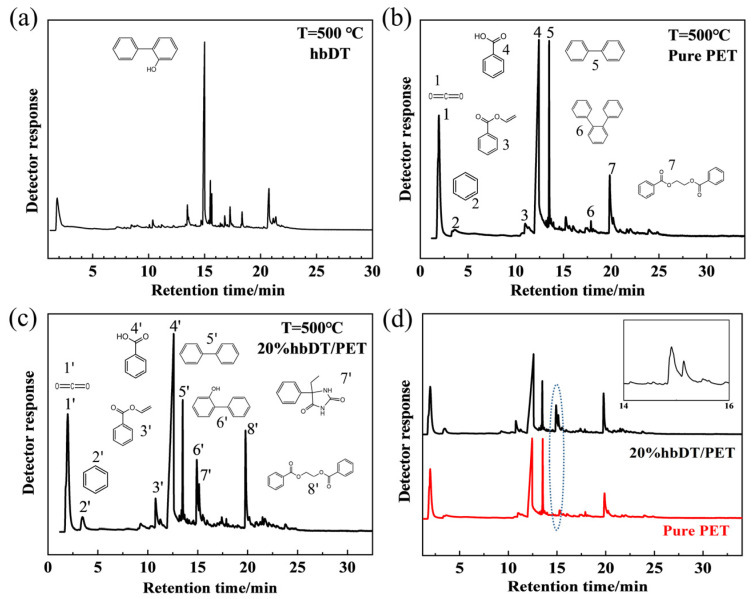
Ion chromatograms of (**a**) hbDT, (**b**) pure PET, (**c**) hbDT/PET, and (**d**) enlarged comparison chromatograms between pure PET and hbDT/PET in PY-GC/MS test.

To further investigate pyrolysis behavior and the flame-retardant mechanism in gas phase, Py-GC/MS was adopted to analyze the volatile products and the results are shown in Figure 8. Figure 8a is the efflux time-relative intensity diagram of the flame retardant hbDT pyrolysis at 450 °C. The results can be seen that the main gas phase volatiles produced during the cracking of hbDT were DOPO and its derivatives, p-benzene, o-phenylphenol, aromatic compounds, CO_2_, etc., in which the strongest peak located at 15 min was assigned to o-phenylphenol [37]. It can be assumed the hbDT underwent anaerobic cleavage during pyrolysis, resulting in the breakage of the P-C bond. The detailed process was as follows: The P-C bond in hbDT was broken first, resulting in the formation of DOPO and DOPO radicals. Then the DOPO were cleaved to produce dibenzofurans and the DOPO radicals were cleaved to o-phenylphenol and phosphorus-containing radicals.

Figure 8b,c shows the efflux time-relative intensity plots for pure PET and 20%hbDT/PET at 500 °C, respectively. As can be seen from the graphs, the cleavage products of pure PET and 20%hbDT/PET had a high degree of overlap. The main products at degradation were carbon dioxide (1), benzene (2), vinyl benzoate (3), benzoic acid (4), biphenyl (5) terphenyl (6) and ethane-1,2-diyl dibenzoate (7). Figure 8d is the enlarged comparison grams between pure PET and hbDT/PET. It is obviously seen that the cleavage product of 20%hbDT/PET had an additional peak located at 15 min, which was contributed to o-phenylphenol (6′) and ethylphenylhydantoin (7′). Based on the Py-GC-MS results of hbDT, the 6′ peak was corresponded to the main product of DOPO and further degradation of the product in the 7′ peak gave rise to nitrogenous refractory gases. The above analysis indicated that the hbDT/PET decomposed to produce DOPO compounds. The presence of o-phenylphenol (6′) suggested that DOPO subsequently decomposes to produce P-containing radicals such as PO· and PO_2_·, which in further captured the H· and ·OH radicals. Meanwhile, the release of flame-retardant gases substantially reduced the surface temperature of the descending polymer and rapidly diluted the flammable gases such as oxygen to make the flame retardants work [38]. The results demonstrated that the DOPO structure in DDP was an important and effective part of the flame retardant effect in the gas phase.

**Figure 9:**
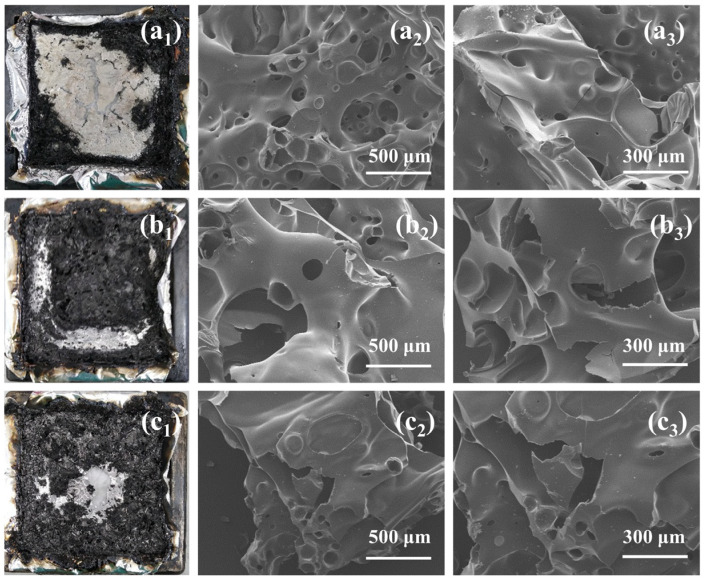
Digital photos and SEM images of the char residues of pure PET (**a_1_**–**a_3_**), 2%hbDT/PET (**b_1_**–**b_3_**), and 5%hbDT/PET (**c_1_**–**c_3_**) after cone calorimeter tests. Subscript 1 is the digital photo, 2 is the SEM images (×60) and 3 is the magnified SEM images (×100).

The surface morphologies of the char residues after cone calorimeter test is another important approach to understand the flame retardant mechanism. The digital photos and SEM images of char residues for pure PET and hbDT/PET composites with different hbDT contents were exhibited in Figure 9. It can be clearly observed that in Figure 9(a_1_–c_1_), there were little residues after burning of pure PET. As the additive amount of hbDT increased, the char residues of the composites increased. The char residue mass of 5% hbDT/PET was ~3.5 times heavier than that of pure PET. The result indicated that the hbDT exerted a protective effect on the cohesive carbon layer and improved the flame retardancy of PET. Figure 9, subscript 2 and 3 are the SEM images of different materials. As can be seen in Figure 9(a_2_,a_3_), the residue of pure PET was a fractured char with lots of cracks and big holes due to the release of combustible volatiles. However, the char surface displayed the continuous, intact, and intumescent outer surface after the incorporation of hbDT. The results illustrated that the addition of hbDT is beneficial to enhancing the thermal stability of the char layer. The inorganic phosphate compounds generated by the decomposition of hbDT have the positive catalytic effect on char formation. Furthermore, the chars covered on the surface of PET act as a protective barrier to prevent the underlying materials from further decomposing and limit the transfer of volatiles as well as heat between gas and condensed phases.

**Scheme 2:**
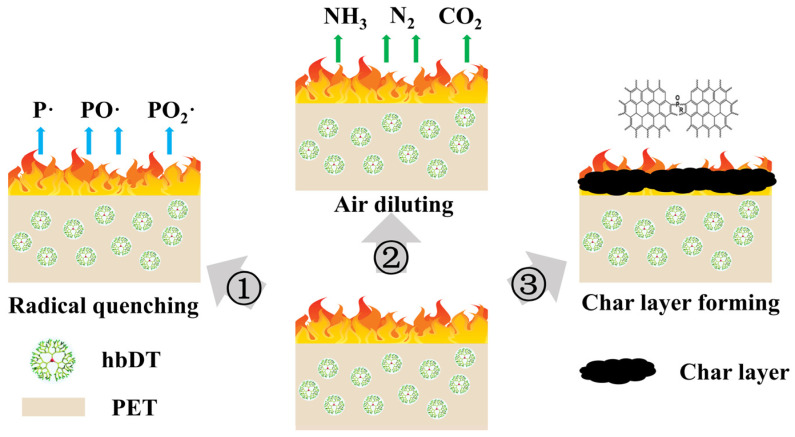
Proposed flame retardant mechanism for hbDT/PET composite.

According to the results of in situ TG-IR, Py-GC-MS, and cone calorimeter tests, the probable flame retardant mechanism of hbDT is proposed in Scheme 2. In the gas phase, hbDT produces volatile phosphorus-containing radicals such as P· and PO·, as well as non-flammable gases containing nitrogen. P· and PO· trap highly reactive radicals, such as HO· and H·, causing the oxidation of hydrocarbons to be weakened or even aborted. At the same time, the release of nitrogen-containing flame-retardant gases removes heat, causing the surface temperature of the polymer drop rapidly. In the condensed phase, the phosphoric acid compounds are produced by the decomposition of hbDT react with the PET matrix to form char containing P-O-C bond [39,40], which is further confirmed by Raman spectra in Figure S3 in supporting information. In the presence of volatile gases, form a dense and swollen char layer, which provides good protection of PET materials. In general, the addition of hyperbranched flame retardants does not affect the processing properties of PET. Due to the rich P and N content resulting from the branched structure, high flame retardancy can be achieved with small additions, and the branched structure helps the formation of cross-linked char layers during combustion, thus improving the flame retardancy of the composite.

## 4. Conclusions

A novel DOPO-based P-N co-efficient hyperbranched flame retardant known as hbDT was successfully synthesized by the esterification between DDP and THEIC monomers. FTIR, ^1^H NMR, and ^13^C NMR were used to characterize the chemical structure of the new flame retardant. hbDT is a polymer molecule with a multi-terminated, highly branched, spherically hyperbranched structure. Due to its unique structure and the synergistic effects of phosphorus and nitrogen, hbDT exhibits high flame retardancy in the PET matrix. According to TGA results, the thermal stabilities and char residues of hbDT/PET composites are improved. In comparison with the pure PET, the LOI value of the 5 wt% hbDT/PET composites is increased from 22.1% to 30.2%. Although molten drops fall in the UL-94 test, the molten drops did not carry fire and could not ignite the skimmed cotton, reaching V-0 level. The flame retardant mechanism of hbDT has been investigated by in situ TG-IR, Cone calorimeter, Py-GC-MS and SEM. The results show that hbDT functions primarily in PET through decomposition of phosphorus-containing radicals to abort the ignition in gas phase, and the release of nitrogen-containing flame-retardant gases removes heat, causing the surface temperature of the polymer drop rapidly. The phosphoric acid compounds produced on the polymer substrate form a stable carbonaceous layer to decrease the concentration of combustible gases and inhibit the heat transfer. This study provided a highly efficient flame retardant additive for the industrial blending method of flame retardant PET, which is of great importance for mass production in the future.

## Figures and Tables

**Table 1:** TG and DTG data of PET and different hbDT/PET composites.

Sample	T_5%_ (°C)	T_max_ (°C)	CY_700 °C_/wt%	T_g_ (°C)
Pure PET	403.2	457.2	8.33	80.6
2%hbDT/PET	408.7	449.0	9.23	81.8
5%hbDT/PET	410.4	452.8	10.39	83.3

T_5%_ defined as the temperature at which 5 wt% weight loss occurred. T_max_ defined as the temperature at maximum weight loss rate. CY_700 °C_ defined as the char yield at 700 °C. T_g_ defined as the glass transition temperature by ISO midpoint.

**Table 2:** LOI values and UL-94 test data of different hbDT/PET composites.

Sample	LOI/%	UL-94	Melt Dripping
Pure PET	22.1	NR	Severe
2%hbDT/PET	28.9	V-2	Moderate
5%hbDT/PET	30.2	V-0	Slow

**Table 3:** The cone calorimeter parameters of pure PET and hbDT/PET composites.

Sample	TTI/s	TpHRR/s	pHRR/kW·m^−2^	THR/MJ·m^−2^	TSP/MJ·m^−2^	AEHC/MJ·kg^−1^	Residue/wt%
Pure PET	49	105	1281.6	73.2	20.5	17.4	2.6
2%hbDT/PET	42	95	719.1	65.7	19.3	16.9	3.5
5%hbDT/PET	45	80	578.1	61.3	19.0	15.8	8.0

## Data Availability

The data presented in this study are available on request from the corresponding author.

## Supplementary Materials

The following supporting information can be downloaded at: https://www.mdpi.com/article/10.3390/polym15030662/s1. Figure S1: Magnified quantitative ^13^C NMR spectra of C in C-N rings from THEIC part in hbDT; Figure S2: SEM and energy dispersive spectroscopy (EDS) spectra of elements mapping distribution for 20%hbDT/PET composites; Figure S3: Raman spectra of char residue from (a) pure PET (b) 2%hbDT/PET and (c) 5%hbDT/PET after cone calorimeter test; Table S1: Comparison of the flame retardant performances of PET composites reported in recent studies.
