# Supplementary material for: Synthesis of DOPO-Based Phosphorus-Nitrogen Containing Hyperbranched Flame Retardant and Its Effective Application for Poly(ethylene terephthalate) via Synergistic Effect
Source: Polymers (Basel). 2023 Jan 28;15(3):662. doi: 10.3390/polym15030662 (PMC9921204; doi:10.3390/polym15030662)
Supplement: Supplementary file 1 [file polymers-15-00662-s001.zip › polymers-2082571-supplementary.pdf]

Supplementary Materials

Synthesis of DOPO-Based Phosphorus-Nitrogen Containing Hyperbranched Flame Retardant and Its Effective Application for Poly(ethylene terephthalate) via Synergistic Effect

Hossamaldin Ahmed Omer Abdalrhem [1], Yueyue Pan [1], Hongda Gu [2], Xiang Ao [1], Xiaohuan Ji [1,*], Xiaoze Jiang [1,*], and Bin Sun [1,*]

1 State Key Laboratory for Modification of Chemical Fibers and Polymer Materials, College of Materials Science and Engineering, Donghua University, Shanghai 201620, China
2 Changshu Polyester Co., Ltd, Changshu 215535, China
* Correspondence: jsjtjxh@gmail.com (X.J.); xiaozejiang@dhu.edu.cn (X.J.); sunbin@dhu.edu.cn (B.S.)

The magnified ^{13}C NMR spectra was illustrated in Fig. S1. The peaks ~148 ppm were ascribed to the C in C-N rings from dendritic units, and the peak at 149.26 ppm was attributed to the C in C-N rings from linear unit and the peaks at 149.71 and 149.64 were came from terminal unit. DB of the hyperbranched polymers has been defined as follows:

$$DB=(D+T)/(D+T+L) \quad (1)$$

Where D, T and L refer to the numbers of dendritic, terminal and linear unites respectively. Due to the area integration of D (2.36), T (0.17) and L (1.38), the DB is calculated to be 0.65.

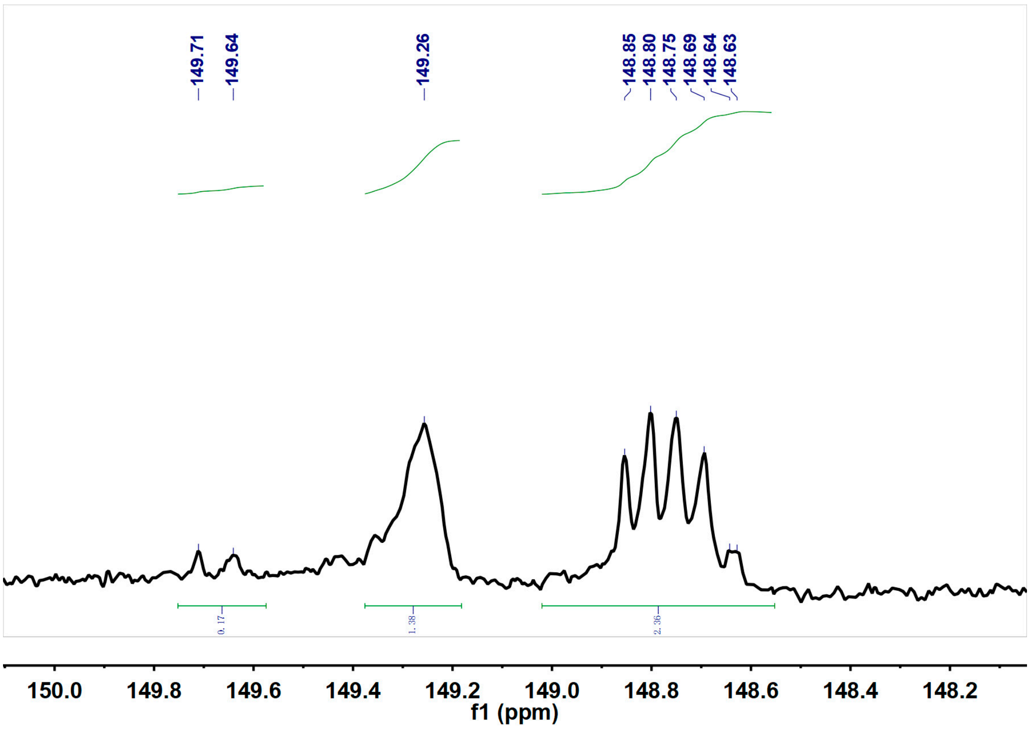

Figure S1. Magnified quantitative ^{13}C NMR spectra of C in C-N rings from THEIC part in hbDT

The Energy Dispersive Spectroscopy (EDS) spectra of hbDT/PET composite were recorded before combustion. The results showed there were P, N, C and O elements are exhibited in the composites and no impurity presented, the spectra are shown in Fig. S2.

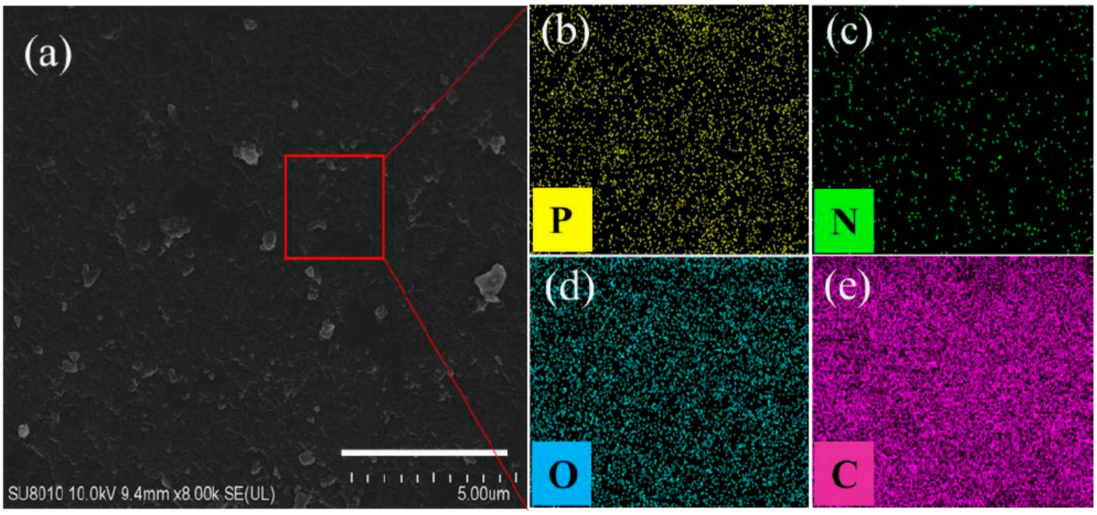

Figure S2. SEM (a) and energy dispersive spectroscopy (EDS) spectra of elements mapping distribution (b) phosphorus, (c) nitrogen, (d) oxygen and (e) carbon for 20%hbDT/PET composites.

In the Raman spectra of carbon materials, the G-peak is generally located at 1580-1600 cm^{-1} and the D-peak at 1350-1580 cm^{-1}. The smaller the ratio of the intensity of the D-peak to the G-peak, i.e. the smaller the ID/IG, the higher the graphitization of the char residue. Raman tests were carried out on the PET and hbDT/PET char residue after cone calorimeter test, the results are illustrated in Fig. S3. The ID/IG of pure PET was 2.5, while the values decreased gradually to 2.04 and 1.82 with 2 wt% and 5 wt% hbDT, respectively. Therefore, the graphitization of the residual char of the hbDT/PET composites increased with the increase of hbDT content, which indicated that the decomposition of hbDT during combustion produced a large amount of phosphoric acid compounds. These phosphoric acid compounds promoted the cross-linking between hbDT and PET matrix to produce char residues and form the char protection, making the flame retardant more efficient.

Based on the Raman and SEM results, it is concluded that in the condensed phase, the phosphoric acid derived from the decomposition of hbDT reacts with PET, followed by the formation of a dense and swollen carbon layer in the presence of refractory and volatile gases.

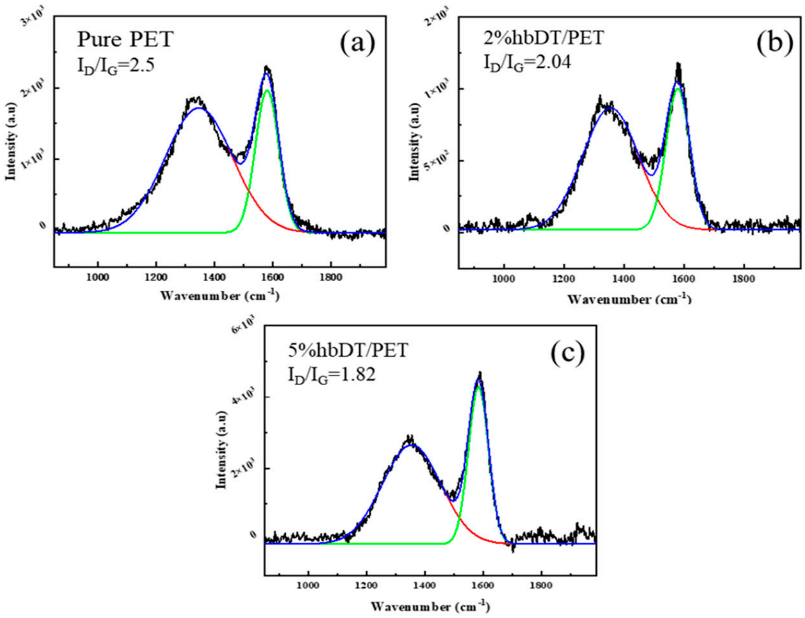

Figure S3 Raman spectra of char residue from (a) pure PET (b) 2%hbDT/PET and (c) 5%hbDT/PET after cone calorimeter test.

Table S1 Comparison of the flame retardant performances of PET composites reported in recent studies

Samples	Preparation method	Flame-retardants	LOI	△pHRR	△THR	Ref
PDPSI/PET	bulk-additive	PDPSI	27.3%	71.2%	29.1%	[1]
BA_nPETs	copolymerization	BA	31.0%	34.0%	16.0%	[2]
PET/FR2.0	bulk-additive	PRS	28.3%	31.1%	13.5%	[3]
PET/DP-DE	bulk-additive	DP-DE	30.5%	-	-	[4]
PET/PZS	bulk-additive	PZS	30.3%	45.5%	12.4%	[5]
PET/HPACP	bulk-additive	HPACP	28.3%	-	-	[6]
PDPSI/Fe_2O_3/PET	bulk-additive	PDPSI, Fe_2O_3	27.9%	66.0%	26.7%	[7]
PET/PNCP/TSCA	bulk-additive	PNCP, TSCA	30.0%	35.7%	9.6%	[8]
PET-MMT/CCS-50/d6	surface treatment	MMT CCS	-	50.2%	27.9%	[9]
hbDT/PET	bulk-additive	hbDT	30.2%	54.9%	16.3%	This work

PDPSI: Poly N-N dimethylene phosphate amidopropyl siloxane. BA: 5-(benzylidene-amino)-isophthalic acid dimethyl ester. PRS: poly (cyclotriphosphazene-resveratrol). DP-DE: 9,10-Dihydro-10-(2,3-dicarboxypropyl)-9-oxa-10-phosphaphenanthrene 10-oxide reacted with ethylene glycol. PZS: poly-(cyclotriphosphazene-co-4,40-sulfonyldiphenol). HPACP: hexa (phenylamino) cyclotriphosphazene.

TSCA: triazine oligoimides containing silicone charring agent. PNCP: hexakis (4-phenoxy) cyclotriphosphazene. MMT: modified montmorillonite. CCS: carboxymethyl chitosan.

1. Y. Peng, B. Xue, Y. Song, et al. Preparation of a novel phosphorus-containing organosilicon and its effect on the flame retardant and smoke suppression of polyethylene terephthalate[J]. *Polymers for Advanced Technologies*, **2019**, 30(5): 1279-1289. https://doi.org/10.1002/pat.4561
2. J.-N. Wu, L. Chen, T. Fu, et al. New application for aromatic Schiff base: High efficient flame-retardant and anti-dripping action for polyesters[J]. *Chemical Engineering Journal*, **2018**, 336: 622-632. https://doi.org/10.1016/j.cej.2017.12.047
3. B. Li, Y. Zhu, X. Wang, et al. Synthesis and application of poly (cyclotriphosphazene-resveratrol) microspheres for enhancing flame retardancy of poly (ethylene terephthalate)[J]. *Polymers for Advanced Technologies*, **2022**, 33(2): 658-671. https://doi.org/10.1002/pat.5548
4. X. Ao, Y. Du, D. Yu, et al. Synthesis, characterization of a DOPO-based polymeric flame retardant and its application in polyethylene terephthalate[J]. *Progress in Natural Science-Materials International*, **2020**, 30(2): 200-207. https://doi.org/10.1016/j.pnsc.2020.01.018
5. Y. Zhu, W. Wu, T. Xu, et al. Preparation and characterization of polyphosphazene-based flame retardants with different functional groups[J]. *Polymer Degradation and Stability*, **2022**, 196: 109815. https://doi.org/10.1016/j.polymdegradstab.2022.109815
6. S. Chen, M. Su, J. Lan, et al. Preparation and properties of halogen-free flame retardant blending modification polyester[J]. *Advanced Materials Research*, **2012**, 463-464: 515-518. https://doi.org/10.4028/www.scientific.net/AMR.463-464.515
7. Y. Peng, M. Niu, R. Qin, et al. Study on flame retardancy and smoke suppression of PET by the synergy between Fe2O3 and new phosphorus-containing silicone flame retardant[J]. *High Performance Polymers*, **2020**, 32(8): 1-12. https://doi.org/10.1177/0954008320914365
8. J. Li, X. Zeng, D. Kong, et al. Synergistic effects of a novel silicon-containing triazine charring agent on the flame-retardant properties of poly(ethylene terephthalate)/hexakis (4-phenoxy)cyclotriphosphazene composites[J]. *Polymer Composites*, **2018**, 39(3): 858-868. https://doi.org/10.1002/pc.24009
9. H. Xie, X. Lai, Y. Wang, et al. A green approach to fabricating nacre-inspired nanocoating for super efficiently fire-safe polymers via one-step self-assembly[J]. *Journal of Hazardous Materials*, **2019**, 365: 125-136. https://doi.org/10.1016/j.jhazmat.2018.10.099
